# Decreasing fluconazole susceptibility of clinical South African *Cryptococcus neoformans* isolates over a decade

**DOI:** 10.1371/journal.pntd.0008137

**Published:** 2020-03-31

**Authors:** Serisha D. Naicker, Ruth S. Mpembe, Tsidiso G. Maphanga, Thokozile G. Zulu, Daniel Desanto, Jeannette Wadula, Nomonde Mvelase, Caroline Maluleka, Kessendri Reddy, Halima Dawood, Motlatji Maloba, Nelesh P. Govender, for GERMS-SA

**Affiliations:** 1 National Institute for Communicable Diseases (Centre for Healthcare-Associated Infections, Antimicrobial Resistance and Mycoses), a Division of the National Health Laboratory Service, Johannesburg, South Africa; 2 School of Pathology, Faculty of Health Sciences, University of the Witwatersrand, Johannesburg, South Africa; 3 Department of Medical Microbiology, Faculty of Health Sciences, University of the Free State, Bloemfontein, South Africa; 4 National Health Laboratory Service, Microbiology Laboratory, Chris Hani Baragwanath Academic Hospital, Johannesburg, South Africa; 5 National Health Laboratory Service, Department of Medical Microbiology, RK Khan Hospital, Durban, South Africa; 6 National Health Laboratory Service, Microbiology Laboratory, Dr George Mukhari Academic Hospital, Pretoria, South Africa; 7 National Health Laboratory Service, Microbiology Laboratory, Tygerberg Academic Hospital, Cape Town, South Africa; 8 National Health Laboratory Service, Microbiology Laboratory, Edendale Hospital, Pietermaritzburg, South Africa; 9 National Health Laboratory Service, Department of Medical Microbiology, Universitas Academic Laboratory Complex, Bloemfontein, South Africa; 10 Division of Medical Microbiology, Faculty of Health Sciences, University of Cape Town, Cape Town, South Africa; Rutgers University, UNITED STATES

## Abstract

**Background:**

Fluconazole is used in combination with amphotericin B for induction treatment of cryptococcal meningitis and as monotherapy for consolidation and maintenance treatment. More than 90% of isolates from first episodes of cryptococcal disease had a fluconazole minimum inhibitory concentration (MIC) ≤4 μg/ml in a Gauteng population-based surveillance study of *Cryptococcus neoformans* in 2007–2008. We assessed whether fluconazole resistance had emerged in clinical cryptococcal isolates over a decade.

**Methodology and principal findings:**

We prospectively collected *C*. *neoformans* isolates from 1 January through 31 March 2017 from persons with a first episode of culture-confirmed cryptococcal disease at 37 South African hospitals. Isolates were phenotypically confirmed to *C*. *neoformans* species-complex level. We determined fluconazole MICs (range: 0.125 μg/ml to 64 μg/ml) of 229 *C*. *neoformans* isolates using custom-made broth microdilution panels prepared, inoculated and read according to Clinical and Laboratory Standards Institute M27-A3 and M60 recommendations. These MIC values were compared to MICs of 249 isolates from earlier surveillance (2007–2008). Clinical data were collected from patients during both surveillance periods. There were more males (61% vs 39%) and more participants on combination induction antifungal treatment (92% vs 32%) in 2017 compared to 2007–2008. The fluconazole MIC_50_, MIC_90_ and geometric mean MIC was 4 μg/ml, 8 μg/ml and 4.11 μg/ml in 2017 (n = 229) compared to 1 μg/ml, 2 μg/ml and 2.08 μg/ml in 2007–2008 (n = 249) respectively. Voriconazole, itraconazole and posaconazole Etests were performed on 16 of 229 (7%) *C*. *neoformans* isolates with a fluconazole MIC value of ≥16 μg/ml; only one had MIC values of >32 μg/ml for these three antifungal agents.

**Conclusions and significance:**

Fluconazole MIC_50_ and MIC_90_ values were two-fold higher in 2017 compared to 2007–2008. Although there are no breakpoints, higher fluconazole doses may be required to maintain efficacy of standard treatment regimens for cryptococcal meningitis.

## Author summary

*Cryptococcus neoformans*, a pathogenic fungal species-complex with an environmental niche, is the most common cause of meningitis among HIV-seropositive adults in sub-Saharan Africa. Fluconazole is recommended in combination with amphotericin B for induction treatment of cryptococcal meningitis and as monotherapy for consolidation and maintenance treatment. Fluconazole is also commonly prescribed to HIV-seropositive individuals for other indications; fluconazole exposure may result in secondary resistance if patients have concurrent active cryptococcal disease. Azole fungicides used in agriculture may potentially drive primary cryptococcal resistance when the fungus is exposed to these fungicides in the environment. We aimed to determine fluconazole MICs in 2017 and compare these values to those obtained in a 2007–2008 South African survey to assess whether fluconazole resistance had emerged in *C*. *neoformans* over a decade. We found that the proportion of isolates with an MIC of ≥16 μg/ml increased from 0% in 2007–2008 to 7% in 2017. MIC_50_ and MIC_90_ values were also two-fold higher in 2017 compared to 2007–2008. These study findings provided evidence for higher fluconazole dose recommendations (in combination with amphotericin B for the induction phase and as monotherapy for consolidation and maintenance phases) in the 2019 Southern African guideline for HIV-associated cryptococcosis.

## Introduction

In South Africa, more than 6500 patients were diagnosed with a laboratory-confirmed first episode of cryptococcal meningitis during 2017, with an estimated incidence risk of 0.1% among HIV-seropositive persons [1]. A third of patients admitted to South African hospitals with cryptococcal meningitis die in hospital [2]. Cryptococcal meningitis is fatal in more than half of treated cases in routine care [3]. Fluconazole monotherapy is not appropriate as a first-line induction-phase treatment but is recommended as an alternative to flucytosine, in combination with amphotericin B. Fluconazole is also recommended for consolidation and maintenance treatment [4]. In an earlier population-based surveillance study of *Cryptococcus neoformans* in Gauteng province, South Africa, fluconazole susceptibility remained largely unchanged between 2002–2003 and 2007–2008. Only 0.6% of incident isolates from 2002–2003 had a fluconazole MIC of ≥16 μg/ml and these isolates also had very low MICs to amphotericin B, voriconazole and posaconazole [5]. In contrast, in a recent Ugandan study, Smith and colleagues documented a substantial increase in fluconazole MICs in 2010–2014 compared to a previous study in 1998–1999. The MIC_50_ and MIC_90_ values were 8 μg/ml and 32 μg/ml respectively in 2010–2014 compared to an MIC_50_ of 4 μg/ml and an MIC_90_ of 8 μg/ml in 1998–1999 [6]. In a systematic review of 21 papers reporting fluconazole MIC distributions for clinical cryptococcal isolates, the median MIC_50_ value increased from 4 μg/ml in 2000–2012 to 8 μg/ml in 2014–2018 [7]. In a US study, 37% of isolates collected between 2001 and 2011 had an MIC ≥16 μg/ml, which the authors considered elevated based on a literature review. This study reported an association between elevated fluconazole MIC and prior azole use [8]. Fluconazole is a broad-spectrum antifungal agent with several indications, including cryptococcal antigenaemia, candidaemia, mucosal candidiasis and common fungal skin infections [9]. Fluconazole is commonly prescribed to HIV-seropositive patients; therefore, fluconazole exposure for other indications could result in development of secondary resistance if patients have concurrent active cryptococcal disease [10]. In agriculture, azole fungicides are used to treat crops. Of 229 pesticides registered in South Africa, 22 are azole-based fungicides [11]. Analogous to the emergence of azole-resistant *Aspergillus fumigatus* [12], it is also possible that cryptococcal strains develop resistance to azoles when exposed to fungicides in the environment and some people develop infections with primarily-resistant strains. The aim of this study was to assess whether there was an increase in fluconazole MIC values in South African clinical cryptococcal isolates since the last survey was performed almost ten years ago.

## Materials and methods

### Study design

Since 2002, the National Institute for Communicable Diseases (NICD) has conducted laboratory-based surveillance for cryptococcosis. A case has been consistently defined as a person diagnosed with cryptococcal disease at any South African laboratory, based on any one of the following positive tests: India ink test on cerebrospinal fluid (CSF), cryptococcal antigen test on blood or CSF and culture of *Cryptococcus* from any specimen. Using a standardised case report form, study nurses at enhanced surveillance sites collected detailed information from participants who met the laboratory case definition. In an earlier survey, Govender et al. had reported that 3/467 (0.6%) clinical *C*. *neoformans* isolates had fluconazole MICs ≥16 μg/ml [5]. In the 2017 survey, we needed to perform antifungal susceptibility testing on at least 220 *C*. *neoformans* isolates to show a difference in prevalence based on 80% power and an alpha of 0.05, if we hypothesized that the proportion of isolates with a fluconazole MIC ≥8 μg/ml had increased from <1% to 5%. In the earlier survey, 280 of 571 first episodes of cryptococcosis from 2007–2008 had been randomly selected from four enhanced surveillance hospitals in Gauteng province; 249 viable isolates were then tested for antifungal susceptibility using the same laboratory methods (with no modifications) as detailed in the section below. We prospectively collected *C*. *neoformans* isolates from 1 January through to 31 March 2017 from persons with a first episode of culture-confirmed cryptococcal disease at 37 enhanced and non-enhanced surveillance hospitals across South Africa. Only incident cases were included and these were defined as participants of any age with first isolation of *C*. *neoformans* from any specimen. We thus compared the antifungal susceptibility profiles of *Cryptococcus* isolates collected during 2 surveillance periods: 1 March 2007 through 28 February 2008 (n = 249, Gauteng province) and 1 January 2017 through 31 March 2017 (n = 229, national [9 provinces]).

### Laboratory methods for 2017 survey

Isolates were received on Dorset transport medium (Media Mage, Johannesburg, South Africa) at NICD, were confirmed as *C*. *neoformans* species-complex by standard phenotypic methods, then stored at -70°C [13]. Canavanine-glycine-bromothymol blue (CGB) agar has a reported 100% specificity for identification of *C*. *neoformans* and was read after 96h of incubation at 30°C [14]. No *C*. *gattii* isolates were identified. Antifungal susceptibility testing was performed on 229 randomly-selected *C*. *neoformans* isolates from first episodes of cryptococcal disease. All phenotypically-confirmed *C*. *neoformans* isolates submitted to NICD during 2017 were labelled consecutively and we randomly selected isolates (based on our required sample size) using a random integer generator (https://www.random.org/integers/). The 229 *C*. *neoformans* isolates were sub-cultured from freezer storage at least twice on Sabouraud agar (Diagnostic Media Products, National Health Laboratory Service [DMP], Johannesburg, South Africa) before antifungal susceptibility testing was performed. We determined fluconazole MICs (range: 0.125 μg/ml to 64 μg/ml) using custom-made broth microdilution panels prepared at NICD and inoculated and read according to Clinical and Laboratory Standards Institute M27-A3 and M60 recommendations [15, 16]. No technical and biological repeats were performed. However, two independent observers blinded to each other’s readings manually read broth microdilution MICs using a reading mirror after 72h of incubation at 35°C. MICs were read at a 50% inhibition endpoint. Each new batch of broth microdilution plates was tested using *Candida parapsilosis* ATCC 22019 and *Candida krusei* ATCC 6258 as quality control strains. MIC values of *C*. *neoformans* isolates from 2017 were then compared to the MIC values of previously tested isolates from 2007–2008 [5]. For isolates with a fluconazole MIC ≥16 μg/ml, we also determined voriconazole, posaconazole and itraconazole MICs by Etest (bioMérieux, Marcy-l'Étoile, France) using RPMI agar (Diagnostic Media Products, NHLS, South Africa). MIC endpoints were also read at 50% inhibition for the above antifungal agents after 72h of incubation at 30°C. A small subset of isolates (n = 20) were randomly selected (using the same random selection mentioned above) from 2007–2008, retrieved from -70°C storage and re-tested using the custom-made fluconazole broth microdilution plates to determine whether MIC values matched values recorded previously [5].

### Statistical analyses

We used chi-square or Fisher’s exact tests to compare proportions and the Wilcoxon rank sum test to compare medians between the two surveillance periods using Stata version 14.1 (StataCorp LP, College Station, Texas, USA). We also determined whether prior hospitalisation (as a proxy for exposure to antifungals) was associated with a fluconazole MIC of ≥16 μg/ml. We performed a sensitivity analysis by varying the cut-offs for an elevated fluconazole MIC (≥8 μg/ml and ≥32 μg/ml) [7].

### Ethics approval

Ethics clearance for this study was obtained from the Human Research Ethics Committee (Medical), University of the Witwatersrand. All data analysed was anonymized.

## Results

**Fig 1:**
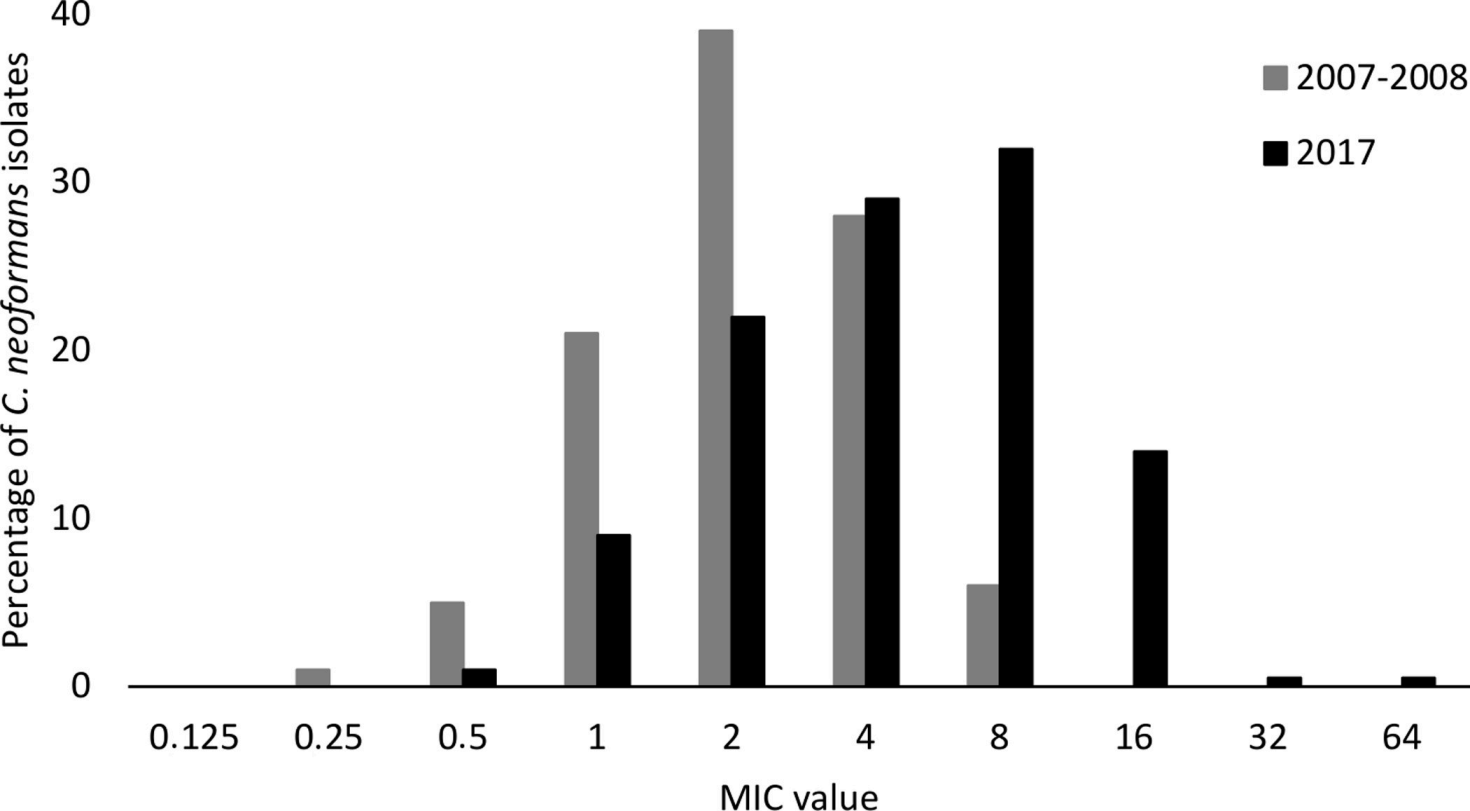
Fluconazole MIC values of cryptococcal isolates between 2 surveillance periods: 1 March 2007–28 February 2008 (n = 249) and January 2017 –March 2017 (n = 229).

In 2007–2008, there were 571 first episodes of cryptococcosis (Gauteng province), 280 of which were randomly selected; 249 viable isolates from these cases were tested for antifungal susceptibility [5]. From January through to March 2017, there were 260 *C*. *neoformans* isolates from first episodes (national survey) that met the inclusion criteria for antifungal susceptibility testing, 229 of which were randomly selected for antifungal susceptibility testing. In 2007–2008, 55% of participants were female with a median age of 35 years (IQR: 30–40 years). In 2017, 59% were male with a median age of 36 years (IQR: 30–43 years). There were more participants on combination induction antifungal treatment (fluconazole and amphotericin B) included in the 2017 survey compared to 2007–2008 (Table 1). The 20 *C*. *neoformans* isolates from the 2007–2008 period that were re-tested on the custom-made broth microdilution plates all had fluconazole MIC values within a double dilution of previously-reported MICs (S1 Table). Table 2 and Fig 1 show the MIC values for the two surveillance periods; in 2007–2008, the geometric mean was 2.08 μg/ml and 4.11 μg/ml in 2017. The MIC_50_ value increased from 1 μg/ml in 2007–2008 to 4 μg/ml in 2017. The MIC_90_ value increased from 2 μg/ml in 2007–2008 to 8 μg/ml in 2017. Voriconazole, itraconazole and posaconazole Etests were performed on 16 of 229 (7%) *C*. *neoformans* isolates with a fluconazole MIC value of ≥16 μg/ml. One isolate had MIC values of >32 μg/ml for voriconazole, itraconazole and posaconazole. The MIC ranges for the other isolates were 0.0004–1.5 μg/ml, 0.012–2 μg/ml and 0.064–8 μg/ml for voriconazole, itraconazole and posaconazole respectively (S2 Table). We found no evidence of an association between prior hospitalisation and a fluconazole MIC of ≥8 μg/ml (crude odds ratio 1.33, 95% CI: 0.59–3.01; p-value = 0.5) and ≥16 μg/ml (crude odds ratio 0.84, 95% CI: 0.17–4.08; p-value = 0.8). Clinical outcome data for participants in both surveillance periods are shown in Table 3. We found an 11% increased unadjusted odds of death among those infected with strains with an MIC ≥8 μg/ml (versus <8 μg/ml), though a 34% reduction to an 86% increase is also compatible with our data (crude odds ratio for death, 1.11; 95% CI: 0.66 to 1.86; p-value = 0.15).

**Table 1:** Comparison of patients with cryptococcosis and antifungal susceptibility results during two surveillance periods: 1 March 2007–28 February 2008 and January 2017 –March 2017.

Characteristic	Number (%)		p value*
	2007–2008 (n = 249)	2017 (n = 204^+^)	
**Sex (n = 453)**	249	204	<0.001
Male	98 (39)	124 (61)	
Female	151 (61)	80 (39)	
**Age, years (n = 450)**	247	203	0.21
Median (IQR)	35 (30–40)	36 (30–44)	
**HIV status (n = 375)**	223	152	0.09
HIV-seropositive	223 (100)	150 (99)	
HIV-seronegative	0 (0)	2 (1)	
**CD4 count, cells/μl (n = 299)**	172	127	0.17
0–50	108 (63)	87 (69)	
51–100	35 (20)	22 (17)	
101–200	24 (14)	10 (8)	
>200	5 (3)	8 (6)	
**Specimen type for positive cryptococcal culture (n = 453)**	249	204	0.39
CSF	218 (88)	172 (84)	
Blood	31 (12)	31 (15)	
Other	0 (0)	1 (1)	
**Antifungal treatment during admission (n = 365)**	235	130	<0.001
Fluconazole alone	121 (51)	5 (4)	
Amphotericin B alone	20 (9)	6 (4)	
Fluconazole and amphotericin B	75 (32)	119 (92)	
No treatment	19 (8)	0 (0)	
**In-hospital outcome (n = 384)**	226	158	0.68
Died	84 (37)	62 (39)	
Survived	142 (63)	96 (61)	

*Wilcoxon rank sum test, chi-squared test or Fisher’s exact test.

^+^ Number of cases after deduplication performed.

**Table 2:** Fluconazole MIC values of cryptococcal isolates collected during 2 surveillance periods: 1 March 2007–28 February 2008 and January 2017 –March 2017.

MIC value (μg/ml)	Number (%) of isolates	
	Feb 2007—Mar 2008 (n = 249)	Jan–Mar 2017 (n = 229)
0.125	0 (0)	0 (0)
0.25	1 (1)	0 (0)
0.5	14 (5)	3 (1)
1	53 (21)	20 (9)
2	97 (39)	50 (22)
4	70 (28)	67 (29)
8	14 (6)	73 (32)
16	0 (0)	14 (6)
32	0 (0)	1 (0.5)
64	0 (0)	1 (0.5)
**Total**	**249**	**229**
**Range**	0.25–8	0.5–64
**MIC**_**50**_	1	4
**MIC**_**90**_	2	8
**Geometric mean**	2.08	4.11

**Table 3:** Association between fluconazole MIC values and clinical outcome of patients with cryptococcosis during two surveillance periods: 1 March 2007–28 February 2008 and January 2017 –March 2017.

MIC value (μg/ml)	Case-fatality ratio, n/N (%)	
	2007–2008	2017
≤2	50/165 (30)	17/66 (26)
2–4	27/70 (39)	22/62 (35)
≥8	7/14 (50)	23/76 (30)
All	84/249 (34)	62/204 (30)

## Discussion

Over the last decade, the fluconazole susceptibility of *C*. *neoformans* isolates from a first episode of disease has decreased overall in South Africa. Fluconazole MIC_50_ and MIC_90_ values were two-fold higher in 2017 compared to 2007–2008. The proportion of isolates with a fluconazole MIC ≥16 μg/ml increased from 0% in 2007–2008 to 7% in 2017. This trend is consistent with studies from Uganda [6], USA [7] and Taiwan [17]. Epidemiological cut-off values currently exist for *C*. *neoformans*: non-typed isolates with an MIC value of ≥16 μg/ml are considered to be non-wild-type [18]. These findings have provided impetus for new fluconazole dosing recommendations from the Southern African HIV Clinicians’ Society (SAHCS) for management of cryptococcal meningitis [19, 20]. Consistent with the antifungal regimens used in the ACTA trial [21], the 2019 SAHCS guideline recommends a fluconazole induction dose of 1200 mg per day (versus 800 mg per day, as previously recommended) in combination with amphotericin B, and a consolidation dose of 800 mg per day (versus previously-recommended 400 mg per day). Based on a MIC_50_ of 4 μg/ml, this will ensure that the area under the curve: MIC ratio (approximated by a daily dose: MIC ratio) is more than 100 for the first 10 weeks of treatment in most cases [7, 22]. Flucytosine should ideally be included in combination with either amphotericin B or fluconazole in the induction-phase regimen for meningitis [21]. Although flucytosine is not registered in South Africa, it is currently available through a clinical access programme [29]. The 2019 guideline also recommends that isolates from the first relapse episode be tested for fluconazole susceptibility rather than waiting for repeated relapses to occur before testing [19, 23]. Fluconazole inhibits the lanosterol 14α-demethylase enzyme encoded by *ERG11* in the ergosterol biosynthesis pathway and targets the same cell processes as amphotericin B [6, 24]. The molecular basis of resistance includes multidrug efflux pump proteins, decreased affinity to target enzymes or overall decreased drug uptake [7]. Heteroresistance which is a phenomenon where strains express a transient adaptation to the drug also occurs [24, 25]. These mechanisms of resistance will need to be investigated further in the few *C*. *neoformans* isolates with a high fluconazole MIC from our study. Secondary resistance may occur in patients with active cryptococcal disease who are exposed to azoles or as a consequence of primary infection with resistant cryptococcal strains from the environment due to triazole fungicide exposure [6]. To support the latter hypothesis, a study has shown that cryptococcal strains exposed to the pesticide tebuconazole caused resistance to fluconazole both in vitro and in vivo [26]. We observed low MICs to voriconazole, posaconazole and itraconazole in most *C*. *neoformans* isolates with a fluconazole MIC ≥16 μg/ml. In a Zimbabwean study, isavuconazole, voriconazole, itraconazole and posaconazole had the most potent in vitro activity against *Cryptococcus* isolates from a cohort of patients with cryptococcal meningitis using the CLSI broth microdilution method [27]. These azoles could be used as alternatives to fluconazole though are associated with much higher costs, more adverse effects, more erratic pharmacokinetics and limited availability in most resource-limited settings [28]. Nasri et al. reported that prior azole exposure was associated with a higher fluconazole MIC in immunocompromised persons especially those with HIV infection. They also found that isolates with a fluconazole MIC ≥16 μg/ml were more likely to be cultured from people with central nervous system complications [7]. We could not determine an association between high fluconazole MIC and prior fluconazole exposure due to data sparsity. Although we did not genotype the *C*. *neoformans* isolates collected over the two surveillance periods, most isolates are likely to belong to the VNI genotype since this was the dominant genotype observed in a random sample of South African cryptococcal isolates over a 5-year surveillance period (Naicker SD, unpublished data). We also compared the fluconazole susceptibility profile of *C*. *neoformans* isolates from a national survey in 2017 to those from Gauteng (provincial) surveillance in 2007–2008 which is a limitation. However, nearly half (100/229) of the isolates tested for antifungal susceptibility in 2017 were from Gauteng province, five (31%) of which had a fluconazole MIC ≥16 μg/ml. We did not determine the molecular mechanism of resistance for the 16 *C*. *neoformans* isolates with a MIC of ≥16 μg/ml.

In conclusion, fluconazole MIC_50_ and MIC_90_ values were two-fold higher in clinical South African *C*. *neoformans* isolates collected in 2017 compared to 2007–2008. The resistance mechanisms of these isolates need to be studied further. These study findings provided evidence for higher fluconazole dose recommendations (in combination with amphotericin B for the induction phase and as monotherapy for consolidation and maintenance phases) in the 2019 Southern African guideline for HIV-associated cryptococcosis. Further efforts are needed to make flucytosine available for induction phase treatment.

## Supporting information

S1 Checklist STROBE checklist. (PDF)

S1 Table Fluconazole MIC values of 20 *C*. *neoformans* isolates from 2007–2008 that were obtained using custom-made broth microdilution plates. (DOCX)

S2 Table MIC values of voriconazole, itraconazole and posaconazole Etest MIC values for 16 *C*. *neoformans* isolates with a fluconazole MIC of ≥16 μg/ml. (DOCX)
