# Supplementary material for: Decreasing fluconazole susceptibility of clinical South African Cryptococcus neoformans isolates over a decade
Source: PLoS Negl Trop Dis. 2020 Mar 31;14(3):e0008137. doi: 10.1371/journal.pntd.0008137 (PMC7108701; doi:10.1371/journal.pntd.0008137)
Supplement: S1 Table — (DOCX) [file pntd.0008137.s002.docx]

Supplementary Table 1: Fluconazole MIC values of 20 *C. neoformans* isolates from 2007-2008 that were obtained using custom-made broth microdilution plates

| **Isolate number** | **Previous MIC value (µg/ml) [5]** | **Repeat MIC value (µg/ml)** |
| --- | --- | --- |
| 1709 | 0.5 | 1 |
| 644 | 2 | 2 |
| 662 | 2 | 4 |
| 6824 | 4 | 2 |
| 6813 | 1 | 2 |
| 6625 | 4 | 4 |
| 5919 | 4 | 2 |
| 6235 | 2 | 2 |
| 6330 | 2 | 2 |
| 390 | 2 | 1 |
| 76 | 1 | 2 |
| 166 | 4 | 4 |
| 384 | 4 | 2 |
| 3164 | 2 | 2 |
| 6681 | 4 | 2 |
| 2049 | 1 | 4 |
| 3612 | 1 | 2 |
| 3635 | 1 | 1 |
| 4264 | 2 | 1 |
| 2421 | 4 | 2 |
