# Supplementary material for: Decreasing fluconazole susceptibility of clinical South African Cryptococcus neoformans isolates over a decade
Source: PLoS Negl Trop Dis. 2020 Mar 31;14(3):e0008137. doi: 10.1371/journal.pntd.0008137 (PMC7108701; doi:10.1371/journal.pntd.0008137)
Supplement: S2 Table — (DOCX) [file pntd.0008137.s003.docx]

Supplementary Table 2: MIC values of voriconazole, itraconazole and posaconazole Etest MIC values for 16 *C. neoformans* isolates with a fluconazole MIC of ≥16 µg/ml

| **Isolate number** | **Voriconazole MIC (µg/ml)** | **Itraconazole MIC (µg/ml)** | **Posaconazole MIC (µg/ml)** |
| --- | --- | --- | --- |
| 53 | 32 | 32 | 32 |
| 88 | 0.38 | 0.75 | 0.125 |
| 93 | 1.5 | 1.5 | 3 |
| 138 | 0.38 | 1.5 | 0.125 |
| 162 | 0.5 | 1 | 0.75 |
| 169 | 1.5 | 2 | 8 |
| 170 | 1 | 1 | 3 |
| 177 | 0.75 | 0.75 | 0.75 |
| 181 | 0.004 | 0.012 | 0.064 |
| 208 | 0.38 | 1.5 | 4 |
| 210 | 0.38 | 2 | 1.5 |
| 211 | 1 | 1.5 | 4 |
| 214 | 0.75 | 2 | 6 |
| 227 | 0.38 | 1 | 0.5 |
| 241 | 0.25 | 0.75 | 0.25 |
| 339 | 0.25 | 1 | 0.5 |
